# ﻿The Dacini fruit flies of Borneo: an annotated checklist with 89 species including three new to science (Tephritidae, Dacinae)

**DOI:** 10.3897/zookeys.1240.148768

**Published:** 2025-06-09

**Authors:** Camiel Doorenweerd, Arthur Y. C. Chung, Andi Maryani A. Mustapeng, Daniel Rubinoff

**Affiliations:** 1 Department of Plant and Environmental Protection Sciences, Entomology Section, College of Tropical Agriculture and Human Resources, University of Hawaiʻi at Mānoa, 3050 Maile Way, Honolulu, Hawaii, 96822-2231, USA University of Hawaiʻi at Mānoa Honolulu United States of America; 2 Sabah Forestry Department, Forest Research Centre, P.O. Box 1407, 90715 Sandakan, Sabah, Malaysia Forest Research Centre Sandakan Malaysia

**Keywords:** *Bactrocera*, biodiversity, biogeography, *Dacus*, Oriental fruit fly, pest, taxonomy, *Zeugodacus*

## Abstract

Fruit flies of the tribe Dacini (Tephritidae) include many agricultural pests, but also crucial pollinators of orchids and other plants. Surveys for Dacini fruit flies were conducted using methyl eugenol, cue lure, and zingerone as male attractants across Sabah, Borneo, Malaysia in 2018 and 2019, in habitats ranging from primary forest in highly protected Conservation Areas, selectively logged forest, secondary forest, and highly disturbed sites. Because 2019 was a mast year with mass fruiting, our surveys of that year collected more than 33,000 flies, compared to just more than 500 flies in 2018 – with similar trapping efforts. Our work adds 46 species to the 43 previously known from Borneo, bringing the total for the island to 89. Three new species are described: Bactrocera (Bactrocera) melanobivittata Doorenweerd, **sp. nov.**, Dacus (Mellesis) danumensis Doorenweerd, **sp. nov.**, and Zeugodacus (Zeugodacus) cataracta Doorenweerd, **sp. nov.** The new species are only found in conservation areas; *B.melanobivittata* is attracted to methyl eugenol and *D.danumensis* and *Z.cataracta* are attracted to zingerone. A discussion on how biogeographic affinities of the species in the checklist support a strong biogeographic boundary across Wallacea is provided, significant records are highlighted, and the relevance of these fly species for protecting agriculture as well as native ecosystems is discussed.

## ﻿Introduction

With a surface area of 743,329 km^2^, Borneo is the third-largest island in the world, behind Greenland and just shy of New Guinea, and was formed through a complex and dynamic geological history heavily influenced by climatological change (Morley 2000). What is now the island of Borneo was part of Sundaland on the Sunda Shelf continental plate up to around 45 Ma, when a warming planet and sea level rise mostly isolated the land mass between 45–25 Ma, only narrowly connected to mainland Asia (Wilson and Moss 1999; De Bruyn et al. 2014). During this period, large parts of what is now the southern part of Borneo were submerged and formed shallow reefs (De Bruyn et al. 2014). In the past 25 Ma, tectonic plate movement in the region has caused an uplift of the entirety of Borneo and included the formation of one of the tallest peaks in Southeast Asia at 4,095 m; Mount Kinabalu near the northern tip of the island (Cottam et al. 2013). This dynamic formation history and intermittent connections with other landmasses gave rise to a diverse array of habitats and the evolution of complex ecosystems, and the present-day islands that were part of the historical region of Sundaland are jointly recognized as one of 25 global biodiversity hotspots with high levels of endemicity (Myers et al. 2000; De Bruyn et al. 2014; Merckx et al. 2015; Neo et al. 2020).

Borneo has not been immune to the consequences of global and regional anthropogenic development. During the last century, extensive habitat loss has been well-documented for plants, birds, and mammals (Scriven et al. 2015; Ocampo-Peñuela et al. 2020). Presently, large areas have been converted to agriculture, primarily palm oil plantations, but there is a patchwork of selectively logged and partially conserved secondary forest that remains (De Bruyn et al. 2014; Chung et al. 2020). Arguably, two of the best-conserved areas of remaining primary tropical rainforest are the Danum Valley (438 km^2^) and Maliau Basin (390 km^2^) in the Malaysian State of Sabah, which have been designated as Conservation Areas since 1984 (Marsh and Greer 1992). Such areas now form crucial refugia on which many species rely for their persistence and these parks offer fleeting opportunities for science to study species before they become extinct. All groups of life on Borneo are still understudied, and dozens of new species of, for example, fish (Hanafi et al. 2022), plants (Rodda and Rahayu 2022), snails (Marzuki et al. 2021), and insects (Tan et al. 2021) are still being discovered and described.

There are more than 1,000 species in the (sub)tropical fruit fly tribe Dacini (Tephritidae: Dacinae) in four genera: *Bactrocera*, *Zeugodacus*, *Dacus*, and *Monacrostichus* (Doorenweerd et al. 2018). The tribe is mostly known for the threat some representatives pose to global agriculture (White and Elson-Harris 1992; Ekesi et al. 2016). However, many of the species in this tribe are also crucial pollinators, particularly for the most diverse orchid genus on the planet, *Bulbophyllum* (Nakahira et al. 2018; Katte et al. 2020; Thawara et al. 2024). The genera *Bactrocera* and *Zeugodacus* are particularly speciose in Southeast Asia and Oceania, with a center of diversity in New Guinea (Drew and Romig 2022). Chua (1998) provided an annotated checklist of Dacini for Malaysia, including the Bornean part; Drew and Romig (2013) broadly studied Dacini in Southeast Asia including Borneo. Between these two major works and several smaller studies, 43 species had been recorded from Borneo; considerably less than the 296 species recorded from New Guinea and adjacent islands (Drew and Romig 2022).

We conducted Dacini fruit fly surveys across Sabah in Malaysian Borneo in 2018 and 2019 using three male attractants; methyl eugenol, cue lure, and zingerone, to better understand Dacini species distributions and their biogeographic connections across Asia as essential data for pest management as well as for conservation. We integrate our data with literature records from across Borneo to present an updated checklist for the island and we describe three species new to science.

## ﻿Materials and methods

### ﻿Fieldwork

**Figure 1.**
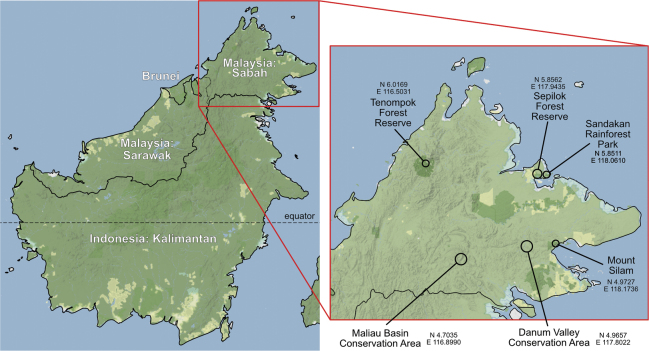
Map of Borneo with the political borders indicated with black lines and the six sampling areas with open circles. The map was generated with the Cartopy package (Met Office 2010) and the Stamen-Terrain tiles from Stamen Design (Stadia Maps 2024); Map tiles © Stadia Maps © OpenMapTiles © OpenStreetMap © Stamen Design.

We conducted surveys for Dacini fruit flies in 2018 and 2019 in six different areas of Sabah, Malaysia (Table 1, Fig. 1). We spaced out trap sites 100–300 m along 4–10 km transects, depending on terrain and trail availability, aiming to cover a variety of habitat types to maximize a diversity of host plants and Dacini species. For the Maliau Basin Conservation Area and Danum Valley Conservation Area we included a transect of several km along the access road before the park entrances which was mainly selectively logged forest. The Tenompok Forest Reserve Area at the foothills of Mount Kinabalu included some traps placed along public roads outside the reserve. The Sepilok Forest Reserve area included the Sepilok Rainforest Discovery Centre, which serves as an arboretum for the general public, as well as an adjacent oil palm plantation which was intermixed with various fruit trees such as papaya and banana. Each trap included a male attractant (lure): either methyl eugenol [ME] (4-allylveratrole CAS No. 93-15-12), cue lure [CL] (4-(p-acetoxyphenyl)-2-butanone CAS No. 3572-06-3), or zingerone [ZN] (4-(4-hydroxy-3-methoxyphenyl)-2-butanone CAS No. 122-48-5), and all three different lure traps were placed at least 3 m apart at each site. Traps were hung on vegetation 1–2 m off the ground with a metal wire. The traps were custom-made from 120-ml plastic cups with two 20-mm holes drilled on either side for the flies to enter and the attractant to disperse. Inside the cup, the attractant and a strip infused with dichlorvos as a killing agent were suspended. For further details on trap design, see Leblanc et al. (2015). Traps were left out in the field for 48 h, after which trapped flies were transferred into tubes with 96% ethanol. Upon return to the lab at the University of Hawaiʻi at Mānoa (**UHIM**) all samples were stored at -20 °C in EtOH. To measure collecting effort, we defined a single ‘trap day’ as a 24-hour period during which all three lure traps were at a particular site (Table 1).

**Table 1.** Survey localities.

Area	Elevation (m)	Survey period	Habitat type	Trap days
Mount Silam	275–640	30.xi–1.xii.2018	Secondary forest	9
Danum Valley Conservation Area	150–340	1–5.xii.2018	Selectively logged and primary forest	134
Tenompok Forest Reserve area	1,265–1,385	7–10.xii. 2018	Secondary forest	140
Maliau Basin Conservation Area	215–950	1–6.viii.2019	Selectively logged and primary forest	180
Sepilok Forest Reserve area	0–75	8–11.viii.2019	Secondary forest and agricultural land	106
Sandakan Rainforest Park	35–75	9–11.viii.2019	Secondary forest	28

### ﻿Species identification

Morphological identification to species-level was made using the keys of Drew and Romig (2016) and Doorenweerd et al. (2024a), and cross-referenced with species descriptions and other literature. Identifications of *B.dorsalis*, *B.occipitalis*, *B.incognita*, *B.raiensis*, *B.borneoensis*, and *B.carambolae* (members of the cryptic *B.dorsalis* clade) were confirmed with genome-wide sequence data; a subset of these samples was used in Doorenweerd et al. (2023a) and San Jose et al. (2023). Identifications of *B.frauenfeldi* are supported by the genome-wide sequence data of Doorenweerd et al. (2023b). The identifications of all other species reported from our surveys are supported by Cytochrome C Oxidase I (COI) sequence data made possible because subsets of samples from the Malaysia surveys were previously used in the global DaciniCOI study of Doorenweerd et al. (2024b). The morphological terminology follows Cumming and Wood (2017) for general Diptera characters and Drew and Romig (2016) for Dacini-specific characters. Voucher specimens used for DNA sequencing are deposited in the University of Hawaii Insect Museum (**UHIM**). Occurrence record data of the trapped specimens, with GPS coordinates, are available as a Global Biodiversity Information Facility (GBIF) dataset: https://doi.org/10.15468/q3a37n.

## ﻿Results

### ﻿Checklist

Our surveys add 46 species to the checklist of Dacini for Borneo (Table 2), which now hosts 89 species in total. The checklist indicates the reference to the first record of each species per political region of Borneo; Sabah now has 79 species recorded, Sarawak 27, Brunei 23, and Kalimantan 26. New lure records are indicated with an asterisk in Table 2, and bolded species names indicate Borneo endemics. A table that further breaks down the species per survey locality within Sabah, as well as the biogeographic affinities of species that also occur outside Borneo, is provided as Suppl. material 1.

**Table 2.** Borneo Dacini checklist.

Species	Lure	Malaysia: Sabah	Malaysia: Sarawak	Brunei	Indonesia: Kalimantan
*Bactroceraabbreviata* (Hardy)	ZN	Chua 1998			
*B.adamantea* Leblanc & Doorenweerd	ZN	This study			
*B.arecae* (Hardy and Adachi)	CL*/ZN*	This study			
*B.bhutaniae* Drew & Romig	CL	This study			
*B.bimaculata* Drew & Hancock	CL	This study			
*B.borneoensis* Doorenweerd & San Jose	ME	This study			
***B.bruneiae*** Drew & Romig	ME			Drew and Romig 2013	Drew and Romig 2013
*B.bullifera* (Hardy)	no known lure		Drew and Romig 2013		
*B.carambolae* Drew & Hancock	ME	Drew and Hancock 1994		Chua 1998	
*B.clarifemur* Leblanc & Doorenweerd	ZN	This study			
*B.cognata* (Hardy & Adachi)	CL	This study			
*B.commensurata* Drew & Romig	ME	This study			
*B.connecta* Leblanc & Doorenweerd	ZN	This study			
*B.dorsalis* (Hendel)	ME	Drew and Hancock 1994		Chua 1998	Drew and Hancock 1994
*B.eurycosta* Drew & Romig	CL	Drew and Romig 2013		Drew and Romig 2013	
*B.flavoscutellata* Lin & Wang	CL	This study			
*B.frauenfeldi* (Schiner)	CL/ZN	This study			Drew and Romig 2013
***B.fulvosterna*** Drew & Romig	CL		Drew and Romig 2013		
*B.fuscitibia* Drew & Hancock	CL/ZN	Drew and Hancock 1994			Drew and Romig 2013
*B.hantanae* Tsuruta & White	CL	This study			
*B.holtmanni* (Hardy)	CL	This study	Drew and Romig 2013		
*B.incognita* Doorenweerd & San Jose	ME	This study			
*B.indonesiae* Drew & Hancock	ME				Drew and Romig 2013
*B.involuta* (Hardy)	CL/ME*	This study			
***B.kalimantaniae*** Drew & Romig	CL	This study			Drew and Romig 2013
***B.kinabalu*** Drew & Hancock	CL/ZN*	Drew and Hancock 1994			
*B.laithieuiae* Drew & Romig	CL	This study			
*B.lata* (Perkins)	CL		Drew and Romig 2013	Drew and Romig 2013	Drew and Romig 2013
*B.lateritaenia* Drew & Hancock	CL(/ZN*)	Drew and Hancock 1994		Drew and Romig 2013	
*B.latifrons* (Hendel)	latilure			Chua 1999	Drew and Romig 2013
*B.limbifera* (Bezzi)	CL	This study	Drew and Romig 2013	Drew and Romig 2013	Drew and Romig 2013
*B.linduensis* Drew & Romig	CL	This study			
*B.malaysiensis* Drew & Hancock	CL	This study			
*B.mediorufula* Drew & Romig	ME	This study	Drew and Romig 2013		Drew and Romig 2013
***B.melanobivittata* sp. nov.**	CL	This study			
*B.melastomatos* Drew & Hancock	CL	This study			Drew and Romig 2013
***B.muiri*** (Hardy & Adachi)	no known lure		Chua 1998		Hardy and Adachi 1954
*B.neocognata* Drew & Hancock	CL	Drew and Hancock 1994		Drew and Romig 2013	Drew and Hancock 1994
*B.neopropinqua* Drew & Hancock	CL	This study			
*B.nigrita* (Hardy)	ME	Chua 1998			
*B.nigrotibialis* (Perkins)	CL	Hardy and Adachi 1954	Drew and Romig 2013	Drew and Romig 2013	Drew and Romig 2013
*B.occipitalis* (Bezzi)	ME	Drew and Hancock 1994		Chua 1999	Drew and Romig 2013
*B.pendleburyi* (Perkins)	ZN	This study			
*B.pernigra* Ito	CL	This study			
*B.propinqua* (Hardy and Adachi)	CL	Hardy and Adachi 1954		Chua 1998	
*B.pseudocucurbitae* (White)	CL	White and Evenhuis 1999	Drew and Romig 2013		Drew and Romig 2013
*B.raiensis* Drew & Hancock	ME	This study			
*B.sembaliensis* Drew & Hancock	ME	This study			
*B.syzygii* White & Tsuruta	ZN	This study			
*B.terminaliae* Drew	CL*	This study			
*B.thailandica* Drew & Hancock	CL	This study		Drew and Romig 2013	
*B.umbrosa* (Fabricius)	ME	This study		Chua 1998	Drew and Romig 2013
*B.unimacula* Drew & Hancock	ME	Drew and Hancock 1994			Drew and Hancock 1994
*B.usitata* Drew & Hancock	CL	This study		Drew and Romig 2013	Drew and Romig 2013
*B.* ‘spMalaysia03’	CL	This study			
*B.* ‘spMalaysia11’	CL	This study			
*B.* ‘spMalaysia15’	ME	This study			
***Dacusleongi*** Drew & Hancock	CL		Drew and Hancock 1998		
*D.longicornis* (Wiedemann)	CL	Hardy and Adachi 1954	Drew et al. 1998	Drew et al. 1998	
*D.ooii* Drew & Hancock	CL		Chua 2010		
*D.sinensis* Wang	ZN	This study			
*D.vijaysegarani* Drew & Hancock	CL	Chua 2010			
***D.danumensis* sp. nov.**	ZN	This study			
*Zeugodacusabnormis* (Hardy)	CL	Drew and Romig 2013			
*Z.apicalis* (de Meijere)	CL	Drew and Romig 2013	Drew and Romig 2013		Drew and Romig 2013
*Z.atrifacies* (Perkins)	CL	This study	Drew and Romig 2013		
*Z.apicofemoralis* (Drew & Romig)	CL				Drew and Romig 2013
*Z.caudatus* (Fabricius)	CL	This study	Drew and Romig 2013	Chua 1998	Drew and Romig 2013
***Z.cataracta* sp. nov.**	ZN	This study			
*Z.cucurbitae* (Coquillett)	CL/ZN	This study		Chua 1998	Drew and Romig 2013
***Z.diaphoropsis*** (Hering)	no known lure		Chua 1998		Hering 1952
*Z.elegantulus* (Hardy)	CL		Drew and Romig 2013		Drew and Romig 2013
*Z.fulvipes* (Perkins)	no known lure	Perkins 1938			
*Z.hoabinhiae* (Drew & Romig)	CL	This study			
*Z.heinrichi* (Hering)	CL/ZN	This study	Drew and Romig 2013		
*Z.hengsawadae* (Drew & Romig)	CL	This study			
*Z.longicaudatus* (Perkins)	CL	Perkins 1938	Drew and Romig 2013		
*Z.longivittatus* (Chua & Ooi)	CL	This study			
*Z.melanofacies* (Drew & Romig)	CL		Drew and Romig 2013		
*Z.nakhonnayokiae* (Drew & Romig)	CL	This study			
*Z.platamus* (Hardy)	CL	Drew and Romig 2013			
***Z.sabahensis*** (Drew & Romig)	CL	Drew and Romig 2013			
***Z.semongokensis*** (Drew & Romig)	CL	Drew and Romig 2013	Drew and Romig 2013		
*Z.signatus* (Hering)	CL	This study			
***Z.speciosus*** (Drew & Romig)	CL	Drew and Romig 2013			
*Z.tau* (Walker)	CL	Drew and Romig 2013	Drew and Romig 2013	Drew and Romig 2013	
*Z.tebeduiae* (Drew & Romig)	CL	Drew and Romig 2013	Drew and Romig 2013	Drew and Romig 2013	
*Z.trichosanthes* (Drew & Romig)	CL		Drew and Romig 2013		
*Z.vinnulus* (Hardy)	CL	This study			
*Z.vultus* (Hardy)	CL	Drew and Romig 2013	Drew and Romig 2013		
*Z.whitei* (Drew & Romig)	CL	Drew and Romig 2013			

### ﻿Identification notes

Cytochrome C Oxidase I (COI) gene sequences for representatives of all the species collected in the 2018 and 2019 surveys were published in Doorenweerd et al. (2024b), except for *B.pernigra* (UHIM identifier ms12595) and *B.platamus* (ms12827), for which those earlier DNA extractions had failed. *Bactroceraterminaliae* (ms12705 and ms12274) was misidentified as *B.quasiinfulata* Drew & Romig in Doorenweerd et al. (2024b); the COI sequences of these specimens are nested in a *B.quasiinfulata* cluster in that study. *Bactroceraterminaliae* was only known from Papua New Guinea and *B.quasiinfulata* is known from Vietnam, Laos, and China; they are distinguished only by differences in the extent of black markings on the mid and fore femur. The only specimen of *Zeugodacushoabinhiae* (ms09131) that we collected was initially misidentified as *Z.diaphorus* (Hendel) in Doorenweerd et al. (2024b); the two are close sister species according to COI sequence data. Representatives of *B.arecae* were included as *B.* ‘spMalaysia07’ in Doorenweerd et al. (2024b) (ms11948 and ms12213). The new record of *B.involuta* is based on a single specimen (ms12105) which matches that species morphologically but it was attracted to methyl eugenol, whereas *B.involuta* is reportedly only attracted to cue lure (Drew and Romig 2013). There are no public reference COI data of *B.involuta* to compare our specimen to. Despite this discrepancy, we maintain our identification and report *B.involuta*, which was previously only known from Sulawesi, as a new record for Borneo.

### ﻿Abundance and diversity

The abundance of species trapped during the surveys in Sabah detailed for each area is provided in Suppl. material 1. The specimen counts for *B.dorsalis*, *B.carambolae*, and *B.occipitalis* (*B.dorsalis* clade) are based on morphology only and may include misidentifications due to the overlapping morphological variation between them (Doorenweerd et al. 2023a). However, representatives from each area were included in the genomic study of Doorenweerd et al. (2023a) to verify presence data. A further 10,153 specimens were identified as “*B.dorsalis* clade” because we could not make a confident species-level assessment based on morphology; these are not included in the species counts per area. The 2018 surveys, on Mount Silam, in Danum Valley Conservation Area, and the Tenompok Forest Reserve area, yielded a total of 502 flies. This was in stark contrast with the 2019 surveys, where we trapped in Maliau Basin Conservation Area, the Sepilok Forest Reserve Area, and Sandakan Rainforest Park, and collected 33,403 flies. The diversity was also greater in the second survey year, but not as dramatically: 31 species in 2018 and 56 species in 2019 (Suppl. material 1). *Bactroceradorsalis* was the most abundant species with 13,612 specimens and was found in all survey areas, followed in abundance by *B.carambolae* with 3,755 specimens. Sixteen species are represented by singletons.

### ﻿Unidentified species

*Bactrocera* ‘spMalaysia04’ (ms08873 and ms08876) and *B.* ‘spMalaysia11’ (ms09142, ms12150, and ms12632) are placed in two clusters, respectively, in the COI tree of Doorenweerd et al. (2024b) and do not closely match any public COI reference sequences. These specimens are all morphologically indistinguishable from *B.carambolae* and *B.incognita*. *Bactrocera* ‘spMalaysia04’ groups as sister to *B.selenophora* Tsuruta & White, and *B.* ‘spMalaysia11’ groups as sister to a cluster that includes *B.pedestris* (Bezzi), *B.silvicola* (May), and various other species. Without further data (i.e., genomic) we refrain from describing these specimens as new species. Three specimens referenced as *B.* ‘spMalaysia03’ (ms08891, ms08911, and ms08871) have two red stripes on the scutum and closely resemble *B.melanobivittata*, *B.ellenriederae* Korneyev, Leblanc, Hauser, General & Gaimari, and *B.youngi* Korneyev, Leblanc, Hauser, General & Gaimari, but the COI sequences do not match any of these species and instead most closely match *B.aethriobasis* (Hardy), from which it is morphologically distinct. Because we do not see sufficient morphological characters to separate the *B.* ‘spMalaysia03’ specimens from the described species with two red stripes on the scutum and we lack genomic data, we refrain from describing this species as new to science as well. Three species were found that differed in morphology from described species, and with divergent COI sequences, that are here described as new to science.

#### ﻿ Bactrocera (Bactrocera) melanobivittata

Taxon classificationAnimaliaDipteraTephritidae

Doorenweerd sp. nov.

47665AA3-6DEA-54A9-A9D0-9D2210832D13

https://zoobank.org/E123C269-BF17-404A-BDE0-16AC2F38950C

Fig. 2

##### Type material.

***Holotype*.** Male. “Malaysia: Sabah. 3–5.xii.2018. Danum Valley access rd. 4.9683"N, 117.8173"E. Methyl eugenol trap FF18Ma065. Leg. D. Rubinoff & C. Doorenweerd. DNA sample ms12171”. Deposited at the University of Hawaii Insect Museum reg. no. UHIM.ms12171. ***Paratype***. One male. “Malaysia, Sabah. 2–4.viii.2019. Maliau Basin: camp Agathis trail. 4.7079"N, 116.8947"E. Methyl eugenol trap FF19Ma036. Leg. D. Rubinoff & C. Doorenweerd. DNA sample ms08978.” Deposited at the Sabah Forestry Department Insect Collection, reg. no. UHIM.ms08978.

##### Differential diagnosis.

The two lateral longitudinal red stripes on the black scutum distinguish *B.melanobivittata* from most other *Bactrocera*. The most similar species is *B.bivittata* Lin & Wang, but *B.melanobivittata* can be distinguished by having a scutum that is almost completely black except for two longitudinal red stripes, and the yellow postsutural vittae. There is intraspecific variation in the amount of black on the scutum in *B.bivittata* (Fig. 3) but the red stripes are obscured in darker colored specimens of *B.bivittata* whereas they are of roughly equal width and clearly contrasting in *B.melanobivittata*. *Bactroceramelanobivittata* can be distinguished from *B.ellenriederae*, which also has two red stripes on the scutum, by the costal band on the wing that ends between vein R_4+5_ and M, while the costal band of *B.ellenriederae* reaches vein M, and *B.melanobivittata* is distinguished from *B.youngi* by having only the anterior lateral corners of tergite IV black, whereas *B.youngi* has the lateral quarters of tergite IV black. The most similar species in Borneo is *B.lateritaenia*, which can be distinguished by having tapering yellow postsutural vittae, which are parallel in *B.melanobivittata*.

##### Molecular diagnostics.

The COI-5P3P sequences of ms12171 and ms08978 are most similar to sequences of *Bactrocerabivittata*, but at 6.5% minimum pairwise difference (Doorenweerd et al. 2024b).

##### Description.

**Figure 2.**
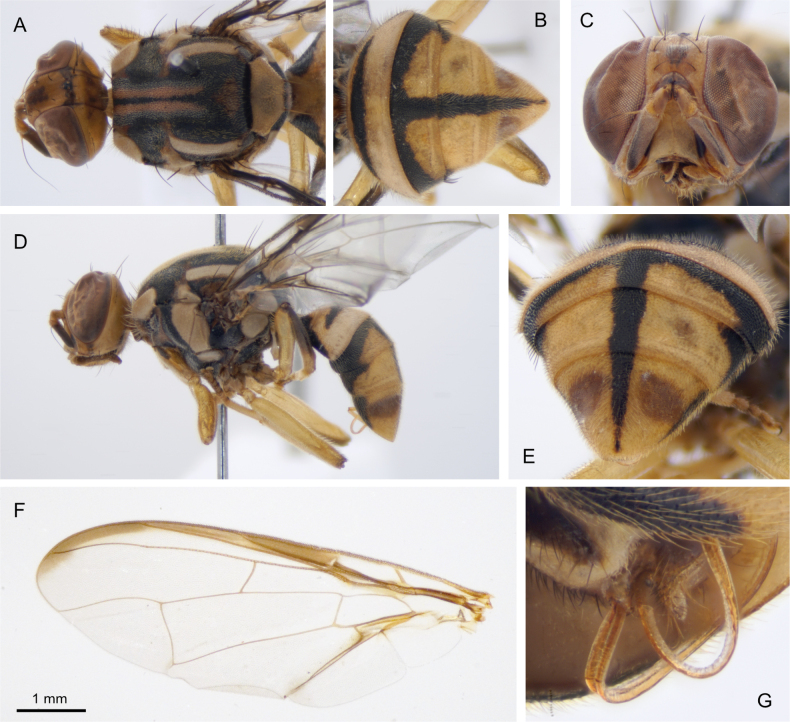
Holotype male of *Bactroceramelanobivittata* sp. nov., UHIM.ms12171 **A** dorsal view of head and thorax **B** dorsal view of abdomen **C** anterior view of the head **D** lateral view of habitus **E** posterior view of abdomen showing the ceromata **F** left wing, dissected and slide mounted **G** detail image of the genitalia showing the surstylus length.

**Male. *Head*** (Fig. 2C). Fulvous with oval black spots in the antennal furrows. Antennal segments fulvous to dark fulvous distally, combined length less than the height of the head. ***Thorax*** (Fig. 2A, B, D, E). Scutum and pleural areas all black with narrow red-brown areas surrounding the yellow markings. Two lateral longitudinal red stripes reach from the anterior of the scutum to half-way of the postsutural yellow vittae. Yellow markings: postpronotal lobes; notopleura; presutural area adjacent to notopleura; postsutural yellow lateral vittae of equal width throughout and reach just past intra-alar seta; mesopleural stripe dorsally wider than notopleuron but does not reach postpronotal lobe; anatergite and katatergite. Scutellum yellow with a narrow black basal band. Setae: one pair scutellar; one pair prescutellar; one pair intra-alar; one pair posterior supra-alar; one pair anterior supra-alar; two pair notopleural and four scapular. ***Abdomen*** (Fig. 2B, D, E, G). Diamond-shaped; terga free; pecten of setae on tergum III present. Dorsal side of abdomen fulvous with a black ‘T’ on segments III–V, the medial line narrows posteriorly to a point on segment V. Anterolateral corners of segment IV black. Ceromata brown. Posterior lobe of the male surstylus short (Fig. 2G). Sternum V with a narrow concavity on the posterior margin. ***Legs*** (Fig. 2D). All femora and tibia fulvous. ***Wings*** (Fig. 2F). Wing length 6.5 mm. Cells *bc* and *c* clear, faint tint in cell *sc.* Costal band confluent with vein R_2+3_ and continues at more or less the same width until just past where vein R_4+5_ reaches the costa. Anal streak absent, vein CuA_2_ and A_1_ merge at ~ 0.8 length of A_1_. Supernumerary lobe not pronounced, inconspicuous. **Female.** Unknown.

**Figure 3.**
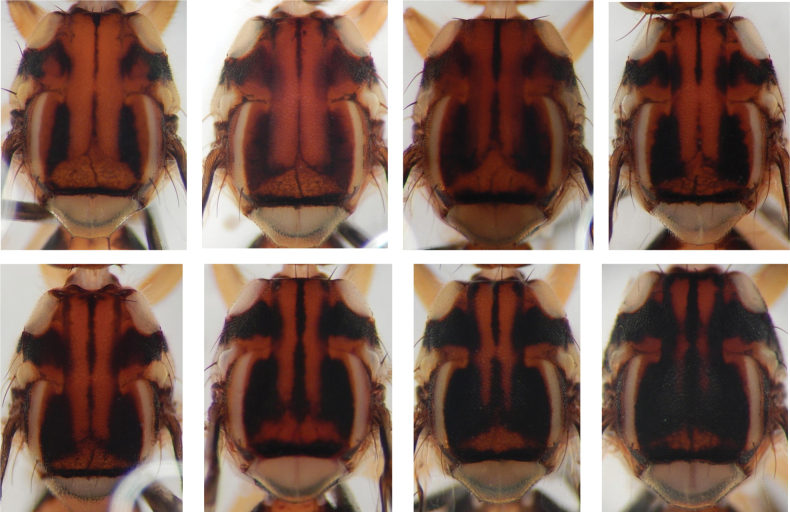
Variation in dorsal scutum coloration of *Bactrocerabivittata*, sister species to *B.melanobivittata* sp. nov. Specimens from China and Laos, photographed in ethanol. University of Hawaii Insect Museum specimen identifiers, from left to right, top to bottom: ms03606, ms01304, ms03607, ms01305, ms03604, ms03609, ms03608, and ms01790.

**Male lure.** Methyl eugenol.

##### Host plant.

Unknown.

##### Etymology.

The species epithet is a compound adjective formed from the Latin *melano*, meaning dark, and its nearest sister species *B. bivittata*, because it has an overall darker appearance of the latter.

##### Comments.

*Bactroceramelanobivittata* was included as *B.* ‘spMalaysia05’ in the DNA barcoding study of Doorenweerd et al. (2024b). It is placed in subgenus Bactrocera based on having a short posterior lobe of the male surstylus, sternum V has a narrow concavity on the posterior margin, lateral postsutural yellow vitta present, medial vitta is absent, one pair of prescutellar setae and the presence of a pair of anterior supra-alar seta.

#### ﻿ Dacus (Mellesis) danumensis

Taxon classificationAnimaliaDipteraTephritidae

Doorenweerd sp. nov.

3A334A53-0B29-5861-BFAE-BFA058163E70

https://zoobank.org/B019FB15-559D-4972-8056-0E3CC6226C94

Fig. 4

##### Type material.

***Holotype*.** Male. “Malaysia: Sabah: Danum valley Tembaling waterfall trail. WGS84 4.9534, 117.8062 3–5.xii.2018 Zingerone trap. Leg. D. Rubinoff & C. Doorenweerd. DNA sample ms08931.” Deposited at the University of Hawaii Insect Museum reg. no. UHIM.ms08931.

##### Differential diagnosis.

The combination of an elongate and petiolate (‘wasp-like’) abdomen, a uniquely shaped postsutural medial yellow vitta that is almost equally wide as long, the wing with a costal band that reaches up to vein R_4+5_ and does not expand apically, and the absence of an anal streak, is unique within Dacini. The most closely related species based on COI sequence data is *Dacussinensis* Wang, from which *D.danumensis* can be morphologically distinguished by having a medial postsutural yellow vitta, which is absent in *D.sinensis*.

##### Molecular diagnostics.

*Dacusdanumensis*COI-5P3P sequences are most similar to sequences of *D.sinensis* at 8.6% minimum pairwise difference (Doorenweerd et al. 2024b).

##### Description.

**Figure 4.**
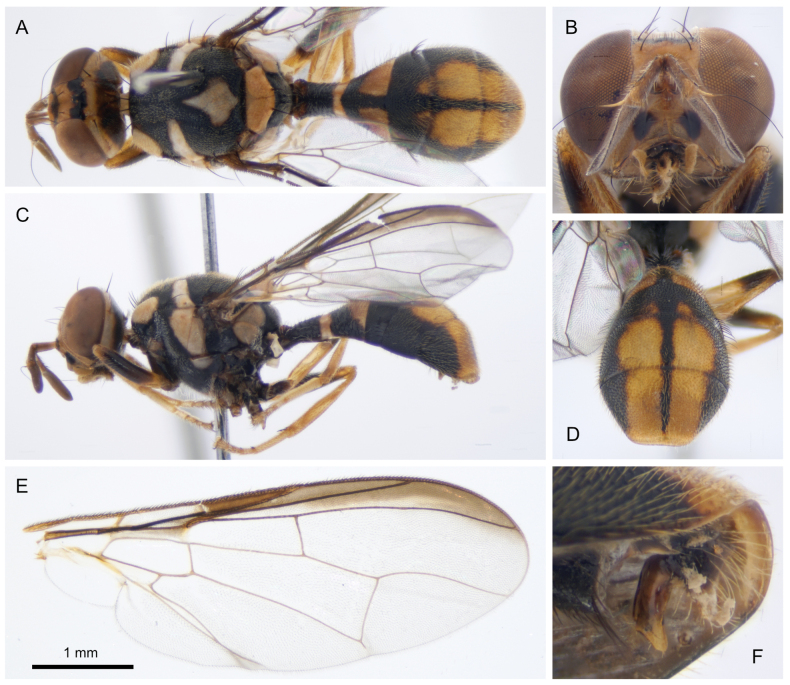
Holotype male of *Dacusdanumensis* sp. n., UHIM.ms08931 **A** dorsal view **B** anterior view of the head **C** lateral view **D** dorso-anterior view of the abdomen showing the ceromata **E** right wing, dissected and slide mounted **F** detail image of the genitalia showing the surstylus length.

**Male. *Head*** (Fig. 4B). Fulvous with two waterdrop-shaped black facial spots in the antennal furrows and a black band connecting both compound eyes across the occiput. Antennae dark fulvous. Combined length of antennal segments greater than the height of the head ***Thorax*** (Fig. 4A, C). Scutum and pleural areas completely black. Yellow markings: postpronotal lobes; notopleura and adjacent presutural area; medial postsutural roughly waterdrop-shaped vitta; mesopleural stripe approx. the width of the notopleuron, anterior margin slightly convex; anatergite, katatergite; scutellum. Setae: one pair scutellar; prescutellar setae absent; one pair intra-alar; one pair posterior supra-alar; one pair anterior supra-alar; two pair notopleural; four scapular. ***Abdomen*** (Fig. 4A, C, D, F). Abdomen shape petiolate with narrow and elongated segment I; terga fused; pecten present on tergum III; posterior lobe of the male surstylus short (Fig. 4F); abdominal sternum V with a narrow concavity on the posterior margin. Tergum I black with yellow posterior band; tergum II yellow with a black T-shape; tergum III with a second black T-shape that covers most of the segment and a medial black line that continuous from tergum II down to the posterior of tergum V; lateral black bands on tergum IV and V. Ceromata yellow and indistinct. ***Legs*** (Fig. 4C). Fore tibia with dark mark on the outer surface that covers most of the tibial length; mid tibia with dark marks on the outer and inner surface that extend from one-third the distal end; hind tibia with distal quarter black. All other leg segments yellow to fulvous. ***Wings*** (Fig. 4E). Wing length 4.9 mm. Cells *bc* and *c* clear, faint tint in cell *sc.* Costal band reaches up to vein R_4+5_, slightly crosses R_4+5_ apically. Anal streak absent, supernumerary lobe not pronounced. **Female.** Unknown.

**Male lure.** Zingerone.

##### Host plant.

Unknown.

##### Etymology.

The species epithet *danumensis* is a noun in apposition that refers to the type locality; the Danum Valley Conservation Area in Sabah, Malaysia.

##### Comments.

This species was included as ‘*Dacus* spnMalaysia01’ in the DNA barcoding study of Doorenweerd et al. (2024b). Placement in subgenus Mellesis is based on lacking prescutellar setae, a slight concavity on the posterior margin of sternum V and its elongate and petiolate abdomen shape.

#### ﻿ Zeugodacus (Zeugodacus) cataracta

Taxon classificationAnimaliaDipteraTephritidae

Doorenweerd sp. nov.

217BC2A4-A422-5A07-BD31-FB265F842C3D

https://zoobank.org/3E638201-3EA4-455D-831C-9BDF84ED3C9C

Fig. 5

##### Type material.

***Holotype*.** Male. “Malaysia: Sabah: Danum Valley Tembaling waterfall trail. WGS84 4.9506, 117.8061 3–5.xii.2018 Zingerone trap. Leg. D. Rubinoff & C. Doorenweerd. DNA sample ms08933.” Deposited at the University of Hawaii Insect Museum reg. no. UHIM.ms08933. ***Paratype***. One male. “Malaysia, Sabah, Danum Valley access road. WGS84 4.9689, 117.8126 3–5.xii.2018 Zingerone trap. Leg. D. Rubinoff & C. Doorenweerd. DNA sample ms08929.” Deposited at the Sabah Forestry Department Insect Collection reg. no. UHIM.ms08929.

##### Differential diagnosis.

The wing pattern of *Zeugodacuscataracta* is strikingly different from any other species of Dacini, with a medial dark band that extends down to midway of cell *dm*, dark marking throughout cell *br*, and three dark smudges in the distal part of the wing. The species is further unique in having vein CuA_2_ and A_1_ merge at ~ 1/2 the length of A_1_, leaving a short cell *cup*, and all antennal segments are remarkably short, combined less than half the height of the head.

##### Molecular diagnostics.

The COI-5P3P sequences of ms08929 and ms08933 are most similar to published sequences of *Zeugodacusscutellaris* (Bezzi, 1913), but at 8.9% minimum pairwise difference (Doorenweerd et al. 2024b).

##### Description.

**Figure 5.**
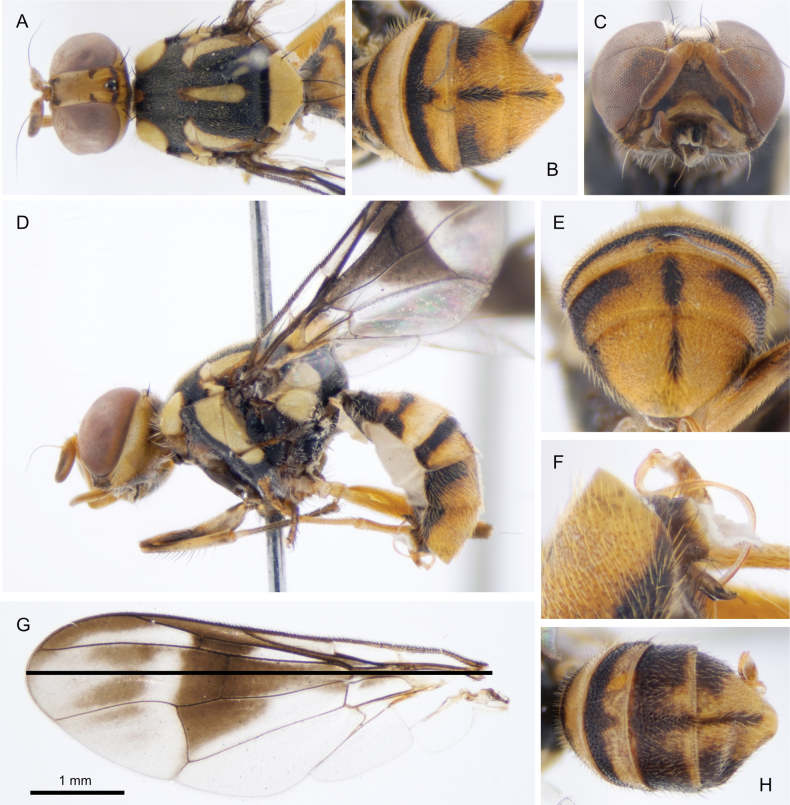
Holotype male of *Zeugodacuscataracta* sp. nov., UHIM.ms08929 **A** dorsal view of head and thorax **B** dorsal view of abdomen **C** anterior view of the head **D** lateral view **E** dorso-anterior view of the abdomen showing the ceromata **F** detail image of the genitalia showing the surstylus length **G** left wing, dissected and slide mounted **H** dorsal view of the abdomen of the paratype (ms08929) showing variation in the abdomen markings.

**Male. *Head*** (Fig. 5C). Fulvous; face dark with indistinct oval black spots in the antennal furrows. Antennal segments dark fulvous and short, combined length less than half the height of the head. ***Thorax*** (Fig. 5A, D). Scutum and pleural areas black with narrow red-brown areas surrounding parts of the yellow markings. Yellow markings: postpronotal lobes; notopleura; presutural area adjacent to notopleura; postsutural yellow lateral vittae that narrow sharply posteriorly and do not reach intra-alar seta; medial elongate postsutural vitta; mesopleural stripe dorsally wider than notopleuron but does not reach postpronotal lobe; anatergite; katatergite. Scutellum yellow with a narrow black basal band. Setae: two pair scutellar; one pair prescutellar; one pair intra-alar; one pair posterior supra-alar; one pair anterior supra-alar; two pair notopleural and four scapular. ***Abdomen*** (Fig. 5B, E, F, H). Oval to diamond-shaped; terga free; pecten of setae on tergum III absent. Posterior lobe of the male surstylus long (Fig. 5F). Abdomen overall fulvous with interrupted black ‘T’ on segments III–V. Anterolateral corners of segment IV black. Paratype with more extensive black lateral markings on tergum IV and V (Fig. 5H). Ceromata yellow and indistinct. ***Legs*** (Fig. 5D). Legs overall fulvous. Outer surface of the fore tibia black, distal third of hind tibia black. ***Wings*** (Fig. 5G). Wing length 5.8 mm. Cells *bc* and *c* clear, faint tint in cell *sc.* Costal band confluent with vein R_2+3_ and continues at more or less the same width until just past where vein R_4+5_ reaches the costa. Anal streak absent, vein CuA_2_ and A_1_ merge at # 1/2 the length of A_1_. A medial dark band extends from the costal band down to midway of cell *dm* and dark marking throughout cell *br.* The distal part of the wing has three dark brown smudges below vein R_4+5_. **Female.** Unknown.

**Male lure.** Zingerone.

##### Host plant.

Unknown.

##### Etymology.

The species epithet *cataracta*, Latin for waterfall, is a noun in apposition that refers to both the peculiar wing pattern that resembles a waterfall going over the edge and the type locality; the Tembaling waterfall trail in the Danum Valley Conservation Area.

##### Comments.

This species was included as *Zeugodacus* ‘spnMalaysia02’ in the DNA barcoding study of Doorenweerd et al. (2024b). The combination of a short cell *cup* and antennae shorter than the height of the head makes accurate systematic placement of this species challenging. Within Dacini, this combination is only known in the monotypic BactrocerasubgenusQueenslandacus; *B.exigua* (May), known from Australia. However, the yellow presutural markings and long posterior lobes of the male surstylus clearly place this species in *Zeugodacus*, and we find placement in subgenus Zeugodacus best fitting and consider the similarity with *B.exigua* paralogous.

## ﻿Discussion

With the new records from our survey, the 89-species Dacini diversity of Borneo is now similar to other areas in Asia where recent surveys have been conducted, such as Sulawesi with 83 species (Doorenweerd et al. 2020) and Vietnam with 93 species (Leblanc et al. 2018). However, it should be noted that our survey method only targets species that respond to the lures that we used, and the true diversity is likely significantly greater. The new Dacini we record for Borneo are likely not recent introductions but previously overlooked native species. Thirteen species (15%) on the checklist are endemic to Borneo. The majority of species, 68 (76%), are also found in mainland SE Asia and/or the Philippines, indicating the strong biogeographic link from the historical region of Sundaland. Eighteen species (20%) are shared with the neighboring island of Sulawesi, part of the biogeographical region of Wallacea (Doorenweerd et al. 2020). Only three species (3%) are also found east of Wallacea, crossing Lydekker’s line (Lydekker 1896) that runs just west of New Guinea: *B.frauenfeldi* - mango fruit fly, *B.umbrosa* - breadfruit fly, and *B.terminaliae*. The first two are common human commensals and may represent semi-recent introductions west across Lydekker’s line (Krosch et al. 2018; Doorenweerd et al. 2023b). Little is known about *B.terminaliae*, which was previously only recorded from New Guinea and no hosts are known (Drew 1989). COI sequence data (Doorenweerd et al. 2024b) as well as morphology suggest a very close affinity of *B.terminaliae* to *B.quasiinfulata*, which is widespread in Southeast Asia and may prove conspecific in further studies. Overall, the biogeographic affinities of the Dacini of Borneo reflect the biogeographic boundary across Wallacea, particularly Lydekker’s line. This is congruent with other studies that found a fauna largely isolated by Lydekker’s line (Michaux and White 1999; Krosch et al. 2012, 2018; Doorenweerd et al. 2020; Starkie et al. 2024). Dacini surveys of the Maluku Islands and other parts of Indonesia, which have been little studied so far, will be crucial to fill further knowledge gaps in the current and historical distribution of Dacini across this region.

Only two species were found in all five survey areas across Sabah: *Bactroceradorsalis* and *B.melastomatos*. *Bactroceradorsalis*, Oriental fruit fly, is the most economically damaging pest within Dacini due to its extreme polyphagy and invasion records spanning continents – but Borneo is considered part of its native range (Clarke et al. 2019). We found it to be the dominant species during our surveys; 40% of all specimens collected were identified as *B.dorsalis* based on morphology. Although this may include some misidentifications as members of the *B.dorsalis* complex cannot all be separated reliably based on morphology alone (Doorenweerd et al. 2023a), our results suggest that *B.dorsalis*, even in its natural environment, is a dominant species in Borneo ecosystems. *Bactroceraborneoensis* and *B.incognita* are two recently described species that were previously confused with *B.dorsalis* (Doorenweerd et al. 2023a) and have undoubtedly been confused in our counts of *B.dorsalis*. It is unclear whether they are pests of commercial crops. We only found *B.borneoensis* – confirmed with genomic data (San Jose et al. 2023) – commonly in the more natural areas of Danum Valley, Maliau Basin, and Tenompok Forest Reserve, suggesting it is native to Borneo and is not a pest species. *Bactroceraincognita* was only revealed with genomic data (Doorenweerd et al. 2023a) on Mount Silam; although it remains unclear if it is native to Borneo, its low abundance does not suggest it is currently a pest of commercial concern. *Bactroceramelastomatos* is genetically indistinguishable from *B.rubigina* and *B.osbeckiae*, both based on COI (Doorenweerd et al. 2024b) and also from whole genome data (Dupuis et al. 2018). The three are distinguished by the extent of black markings on the scutum, with *B.rubigina* completely or almost completely red, *B.osbeckiae* with ~ 50% red and ~ 50% black, and *B.melastomatos* mostly black (Drew and Romig 2013). This small group of species is common throughout Southeast Asia and requires further taxonomic study, but there are no records of any of them being economically damaging.

Although our surveys were designed to collect as many species as possible and not to compare diversity or abundance between sites, there are some notable trends in our results. The Maliau Basin was the most diverse area during our surveys with 45 species, followed by the Sepilok Forest Reserve with 28 species and Danum Valley with 27 species. The Tenompok Forest Reserve, at the base of Mount Kinabalu, only had five species but at 1,265–1,385 meters in elevation this cooler area was expected to be less diverse for a (sub)tropical group of flies. The large difference in diversity between the two most undisturbed areas that we surveyed, Maliau Basin and Danum Valley, is likely due to the climatological events that occurred between the two survey years and may not reflect habitat quality. In 2018, when we visited Danum Valley our surveys yielded a total of 502 flies, whereas the 2019 surveys, when Maliau Basin was visited, yielded 33,403 flies. The trapping efforts between both years were similar; this large difference in fly abundance is possibly a consequence of 2019 being a mast year. Mast years in Borneo occur every 4–10 years after a period of drought and result in > 80% of all the angiosperms flowering simultaneously, followed by a super abundance of fruit (Kettle et al. 2010), and evidently, extremely high fruit fly densities. Little is known about the importance of mast years for the functioning of tropical ecosystems (Appanah 1993), but our results suggest that they have massive impacts on insect population dynamics that warrant further study.

Two polyphagous pest species were not as widespread as might have been expected: *B.frauenfeldi* and *Z.cucurbitae*. *Bactrocerafrauenfeldi*, the mango fruit fly, is a common pest that is widespread across New Guinea and surrounding islands, the Solomon Islands, and northern Australia. Borneo is the (present) northwestern limit of its distribution. *Zeugodacuscucurbitae*, melon fly, is a common pest of cucurbits and other fruits and is believed to be native to Southeast Asia but has invaded Africa and several Pacific Islands (De Meyer et al. 2015). In our surveys, *B.frauenfeldi* and *Z.cucurbitae* were only found in Sandakan Rainforest Park and the Sepilok area and possibly represent recent introductions that have not spread outside this region and could potentially be controlled to prevent further spread. Species not native to Borneo have the potential to become invasive causing agricultural damage and, in natural forests, disrupting both native orchid pollination as well as fruit production and dispersal. However, we cannot rule out that *B.frauenfeldi* and *Z.cucurbitae* may already be more prevalent in agricultural areas across Borneo, as our sampling mostly focused on less disturbed regions. Another polyphagous pest species, *Z.tau*, was only found in the Danum Valley and Maliau Basin Conservation Areas; suggesting that Borneo is (part of) its native range. It is widespread in Southeast Asia and causes significant economic losses of particularly cucurbits (Liu and Ji 2024), but we did not encounter it outside the natural areas or in numbers comparable to pests like *B.dorsalis* and *B.carambolae*.

Continued Dacini surveys across Borneo will undoubtedly bring more species records and better resolution to current species’ distribution and movement over time. Such information provides essential background for pest management in agriculture as well as management of natural resources. Although more labor intensive, rearing fruit flies from collected fruit in particular would be invaluable in providing new host records, and recording fly species that are not attracted to any of the attractants used during surveys. Further surveys of Sarawak, Kalimantan, and Brunei are likely to yield more undescribed species and new island records as these areas have not been surveyed extensively in recent years, and never with zingerone as a male attractant. Undescribed species can be expected particularly in undisturbed areas; the three newly described species from our study were only found in the nature preserves of Maliau Basin and Danum Valley.

## Supplementary Material

XML Treatment for Bactrocera (Bactrocera) melanobivittata

XML Treatment for Dacus (Mellesis) danumensis

XML Treatment for Zeugodacus (Zeugodacus) cataracta

## ﻿Additional information

### Author ORCIDs

Camiel Doorenweerd https://orcid.org/0000-0002-0418-4439

Arthur Y. C. Chung https://orcid.org/0000-0002-9529-4114

Andi Maryani A. Mustapeng https://orcid.org/0000-0001-5252-4226

Daniel Rubinoff https://orcid.org/0000-0002-2732-3032

### Data availability

All of the data that support the findings of this study are available in the main text or Supplementary Information.

## Supplementary materials

Supplementary material 1 Borneo Dacini checklist per park Data type xlsx Explanation note Excel spreadsheet with the species found in the different political regions of Borneo, the Sabah areas where they were found in the 2018–2019 surveys, and where they are found outside of Borneo. https://binary.pensoft.net/file/1348698This dataset is made available under the Open Database License (http://opendatacommons.org/licenses/odbl/1.0/). The Open Database License (ODbL) is a license agreement intended to allow users to freely share, modify, and use this Dataset while maintaining this same freedom for others, provided that the original source and author(s) are credited.Camiel Doorenweerd, Arthur Y. C. Chung, Andi Maryani A. Mustapeng, Daniel Rubinoff
